# An S-Type Anion Channel SLAC1 Is Involved in Cryptogein-Induced Ion Fluxes and Modulates Hypersensitive Responses in Tobacco BY-2 Cells

**DOI:** 10.1371/journal.pone.0070623

**Published:** 2013-08-12

**Authors:** Takamitsu Kurusu, Katsunori Saito, Sonoko Horikoshi, Shigeru Hanamata, Juntaro Negi, Chikako Yagi, Nobutaka Kitahata, Koh Iba, Kazuyuki Kuchitsu

**Affiliations:** 1 Department of Applied Biological Science, Tokyo University of Science, Noda, Chiba, Japan; 2 Research Institute for Science and Technology, Tokyo University of Science, Noda, Chiba, Japan; 3 School of Bioscience and Biotechnology, Tokyo University of Technology, Hachioji, Tokyo, Japan; 4 Department of Biology, Faculty of Science, Kyushu University, Fukuoka, Japan; School of Medicine and Health Sciences, University of North Dakota, United States of America

## Abstract

Pharmacological evidence suggests that anion channel-mediated plasma membrane anion effluxes are crucial in early defense signaling to induce immune responses and hypersensitive cell death in plants. However, their molecular bases and regulation remain largely unknown. We overexpressed Arabidopsis *SLAC1*, an S-type anion channel involved in stomatal closure, in cultured tobacco BY-2 cells and analyzed the effect on cryptogein-induced defense responses including fluxes of Cl^−^ and other ions, production of reactive oxygen species (ROS), gene expression and hypersensitive responses. The SLAC1-GFP fusion protein was localized at the plasma membrane in BY-2 cells. Overexpression of *SLAC1* enhanced cryptogein-induced Cl^−^ efflux and extracellular alkalinization as well as rapid/transient and slow/prolonged phases of NADPH oxidase-mediated ROS production, which was suppressed by an anion channel inhibitor, DIDS. The overexpressor also showed enhanced sensitivity to cryptogein to induce downstream immune responses, including the induction of defense marker genes and the hypersensitive cell death. These results suggest that SLAC1 expressed in BY-2 cells mediates cryptogein-induced plasma membrane Cl^−^ efflux to positively modulate the elicitor-triggered activation of other ion fluxes, ROS as well as a wide range of defense signaling pathways. These findings shed light on the possible involvement of the SLAC/SLAH family anion channels in cryptogein signaling to trigger the plasma membrane ion channel cascade in the plant defense signal transduction network.

## Introduction

Plants respond to pathogen attacks by activating a variety of immune responses, which restrict pathogen growth at the site of infection [1]. These responses are initiated by recognition of signal molecules/elicitors derived from microbes/pathogens or damaged plant tissues [2].

Upon recognition of the signals, plant cells activate a widespread signal transduction network involving second messengers to trigger inducible immune responses. Characteristic early signaling events include membrane depolarization, plasma membrane effluxes of anions and K+, cytosolic Ca2+ rise, pH changes (cytosolic acidification/extracellular alkalinization), production of reactive oxygen species (ROS), and activation of the mitogen-activated protein kinase (MAPK) cascade [3]–[9]. These initial responses are followed by the synthesis of phytoalexins, vacuolar collapse, hypersensitive cell death, and activation of pathogenesis-related (PR) genes [10]–[12]. These downstream events are often prevented when anion release through the plasma membrane is compromised by several anion channel blockers [13], [14], suggesting the importance of anion release through the plasma membrane in the induction of these responses. Indeed, several studies have reported that in various types of mammalian cells, activation of plasma membrane Cl^−^ channels is an early prerequisite to apoptotic events including cell shrinkage, cytochrome c release, and programmed cell death [15], [16]. These results highlight the involvement of anion release mediated by anion channels located on the plasma membrane in early defense signaling processes. However, the molecular bases for the anion effluxes and its regulation in immune responses remain largely unknown.

Several types of anion channels differing in their voltage dependence, kinetic properties, and anion selectivity have been characterized, mostly by electrophysiological techniques in plants [17]–[19]. Recently, molecular and electrophysiological studies have shown that Arabidopsis SLAC1 is required for anion channel activity in the plasma membrane of guard cells and are more permeable to Cl^−^ than malate [17], indicating that SLAC1 functions as a slow-type (S-type) anion channel located at the plasma membrane. This channel, activated by the stress hormone abscisic acid (ABA), ozone, and CO_2_, is involved in the early steps leading to stomatal closure [20]–[22]. Four orthologs of *SLAC1*, *SLAH1-4* have been identified in Arabidopsis, which constitute the SLAC/SLAH family [20]. However, physiological roles of any SLAC/SLAH family proteins in plant immunity remain totally unknown.

Cryptogein, a 10-kDa proteinaceous elicitor produced by the pathogenic oomycete *Phytophthora cryptogea*, induces a variety of immune responses such as membrane potential changes, extracellular alkalinization, ion fluxes including Ca2+, production of ROS and nitric oxide, MAPK activation, defense-related gene expression, and induction of hypersensitive cell death accompanied by vacuolar collapse in tobacco plants and suspension-cultured cells [4], [11], [23]–[26]. Recent transcriptomic and proteomic analyses have revealed many components involved in the cryptogein signaling network in tobacco [27], [28]. Cryptogein also triggers anion efflux, and pharmacological analyses with anion channel inhibitors including DIDS, 4,4′-diisothiocyanostilbene-2,2′-disulfonic acid, suggest importance of the anion efflux mediated by the plasma membrane anion channels in induction of cryptogein-induced responses in tobacco BY-2 cells [4]. Cryptogein signaling in BY-2 cells thus provide an excellent model system for understanding the roles of anion channels in plant immunity.

We here investigated the effects of overexpression of Arabidopsis SLAC1, an S-type anion channel, on various cryptogein-induced responses in BY-2 cells. SLAC1 expression enormously enhanced cryptogein-induced hypersensitive responses, such as ion fluxes, ROS production, defense-related gene expression, and hypersensitive cell death. Possible physiological function of the SLAC/SLAH family anion channels is discussed.

## Materials and Methods

### Plant material, transformation and selection of transformed cell lines, and growth conditions

The tobacco BY-2 (*Nicotiana tabacum* L. cv. Bright Yellow-2) cell suspension was grown in modified Linsmaier and Skoog (LS) medium as reported [29], and agitated on a rotary shaker at 100 rpm at 25°C in the dark and used 1 week after subculture. To overexpress *SLAC1* in both BY-2 cells, *SLAC1* cDNA [20] was cloned into a Ti-based vector pBI121 downstream of a CaMV *35S* promoter.

Transformation of BY-2 cells was carried out as described in [30] with minor modifications as follows: 4 ml of 3-day-old exponentially growing culture was transferred to a 90 mm Petri dish and incubated at 28°C with 100 µl of fresh overnight-culture of *Agrobacterium tumefaciens* pGV2260 containing the binary vector pBI121. After 48-h co-cultivation, tobacco cells were washed and plated onto LS agar medium containing kanamycin (100 µg ml^−1^) and claforan (100 µg ml^−1^) (Sanofi Aventis Co., Tokyo, Japan). Every 3 to 4 weeks, transformants were selected and transferred onto fresh medium for continued selection.

### Chemicals

DIDS and FM4-64 were obtained from Life technologies (Carlsbad, CA, USA). DPI (diphenylene iodonium), K-252a, and luminol were purchased from Wako Pure Chemical (Osaka, Japan).

### Expression and purification of cryptogein

*Pichia pastoris* (strain GS115) carrying the plasmid pLEP3 was used for cryptogein production [4]. Cryptogein was produced according to O′Donohue et al. [31] and was dissolved in distilled water. Cryptogein concentration was determined using UV spectroscopy employing extinction coefficients of 8306 M^−1^cm^−1^ at 277 nm [32].

### Measurement of pH and [Cl^−^]

To monitor pH of the culture medium, aliquots of cells (30 g fresh weight) that had been subcultured for 3 d were transferred to 30 ml of fresh culture medium lacking KH_2_PO_4_. For extracellular Cl^−^ concentration, aliquots of cells (30 g fresh weight) that had been subcultured for 7 d were transferred to 30 ml of fresh culture medium replacing CaCl_2_ with CaSO_4_. After a 3 h equilibration period on a gyratory shaker (160 rpm, 25°C) in open 100-ml vials, the pH of the culture medium was measured by the compact pH meter (Model AS-212, Horiba, Kyoto, Japan) according to the manufacturer's protocol. [Cl^−^] of the culture medium was measured with a pH/Ion meter (Model F-53, Horiba) with a combination electrode sensitive to Cl^−^ (Model 8002, Horiba).

### Measurement of H_2_O_2_ in tobacco BY-2 cells

BY-2 cells (3 day after subculture) were used for the ROS production assay. To monitor initial H_2_O_2_, cells were washed and resuspended in 5 mM MES buffer (pH 8.0) containing 175 mM mannitol, 0.5 mM CaCl_2_, and 0.5 mM K_2_SO_4_. After a 3 h equilibration period on a gyratory shaker (160 rpm, 25°C), 0.462 mM luminol and 5 mM Tris buffer (pH 8.0) was added. Fifteen minutes after luminol application, H_2_O_2_-dependent chemiluminescence was monitored with a luminometer (Lumicounter 2500, Microtech Nition, Chiba, Japan), in which the culture tube rotates 17 revolutions every 3 s clockwise and counterclockwise in turn, and agitates the cells [4].

To detect prolonged H_2_O_2_ production, an aliquot (25 µl) of medium was mixed in a 96-well microtiter plate with 150 µl 50 mM Tris-HCl (pH 8.0) and 25 µl 0.462 mM luminol in 50 mM Tris-HCl, pH 8.0. Potassium ferricyanide (25 µl, 11.76 mM) was added, and H_2_O_2_-dependent chemiluminescence was recorded for 15 s using a luminometer (MicroLumat Plus LB96V, Berthold Technologies, Bad Wildbad, Germany).

To quantify the effects of inhibitors on ROS production, the peak intensity of luminol chemiluminescence was compared and relative luminescence levels in the control were standardized as 100%.

### Cell death assay with Evans blue

An aliquot of the cell suspension (50 mg fresh weight in 0.5 ml) was incubated with 0.05% Evans blue (Sigma, St. Louis, MO, USA) for 10 min and washed to remove unabsorbed dye. The selective staining of dead cells with Evans blue depends on extrusion of the dye from living cells via an intact plasma membrane [33]. Cell death was quantified by counting stained cells (250–300 cells per each treatment) using a microscope.

### MTT reductase activity

An aliquot of the cell suspension (50 mg fresh weight in 0.5 ml) was incubated with 0.2 ml of 12 mM MTT (3-(4, 5-dimethyl-2-thiazolyl)-2, 5-diphenyl-2H tetrazolium bromide) solution (DOJINDO, Tokyo, Japan) for 0.5 h at 28°C. The supernatant was removed by centrifugation and 1 ml of acidic isopropanol (40 mM HCl) was added to stained cells, followed by incubation at 60°C for 15 min to elute the dye from the cells. *A*_595_ was measured to measure the reductase activity.

### Reverse transcriptase (RT)-PCR analysis

Total RNA was isolated using Sepasol (Nacalai tesque, Kyoto, Japan) according to the manufacturer's protocol and quantified with a spectrophotometer. First-strand cDNA was synthesized from 3 µg total RNA with an oligo-dT primer (Life technologies) and reverse transcriptase (Promega, Fitchburg, WI, USA). PCR amplification was performed with an initial denaturation at 95°C for 3 min followed by the indicated number of cycles of incubations at 94°C for 30 s, 55°C for 90 s, and 72°C for 1 min, and a final extension at 72°C for 10 min using Arabidopsis *SLAC1*-specific primers (*SLAC1*-F 5′-GCCATTAGCGTACCTCCCAT-3′, *SLAC1*-R 5′- GCAGATATTTTCTTCGCCAG-3′). *EF-1α* (*EF1α*-F 5′-ATTCAAGTATGCCTGGGTGCTTGAC-3′, *EF1α*-R 5′-TTCGTTGTCGAGGACCATGC-3′) was used as a quantitative control. Aliquots of individual PCR products were resolved by agarose gel electrophoresis and visualized by ethidium bromide staining using a UV light.

### Real-time RT-PCR quantification

Real-time RT-PCR assays were performed as described by Kurusu et al. [34]. First-strand cDNA was synthesized from 2 µg of total RNA using an oligo-dT primer (Life technologies) and reverse transcriptase (Promega). Real-time PCR was performed using an ABI PRISM 7300 sequence detection system (Life technologies) with THUNDERBIRD SYBR qPCR Mix (TOYOBO, Osaka, Japan) and gene specific primers. The following PCR primers were used: *Hsr203j*-RealF, 5′-GTCTTATCGGAGCAAATCGGAGTTAG-3′; *Hsr203j*-RealR, 5′-CTCCCATCGGACATGTTATTGG-3′; *HIN1*-RealF, 5′-GAGGGTCACAAGAATACTAGCAGC-3′; *HIN1*-RealR, 5′-CGCATGTAAAGCTTCACTTCCATCTC-3′; *NtRbohD*-RealF, 5′-GCGGCAAGTCCAATGATGA-3′; *NtRbohD*-RealR, 5′-GAATCTTCGCGGACATCGA-3′; *NtActin*-RealF, 5′-GGGTTTGCTGGAGATGATGCT-3′; *NtActin*-RealR, 5′-GCTTCATCACCAACATATGCAT-3′ *PR1a*-RealF, 5′-GTGGGTTAGCGAGAAGGCTA-3′; *PR1a*-RealR, 5′-ACTTTGGCACATCCGAGTCT-3′; *AtTUB2*-RealF, 5′-ATTCCCCCGTCTTCACTTCT-3′; *AtRbohD*-RealF, 5′- CCTCAACAACACCACCTCCT-3′; *AtRbohD*-RealR, 5′- GTAAGAGGCCGTTGGAATCA-3′; *AtTUB2*-RealF, 5′-ATTCCCCCGTCTTCACTTCT-3′;*AtTUB2*-RealR, 5′-CACATTCAGCATCTGCTCGT-3′;. Relative mRNA abundances were calculated using the standard curve method and normalized to corresponding *NtActin* and *AtTUB2* gene levels. Standard samples of known template amounts were used to quantify PCR products.

### Intracellular localization of GFP fusion proteins

The green fluorescence protein (GFP) sequence derived from sGFP [35] was fused to the C terminus of Arabidopsis *SLAC1* cDNA and was cloned into a transient assay vector pUC19 downstream of a CaMV *35S* promoter. The original sGFP plasmid was used as a control. Protoplasts were prepared from 3 d BY-2 cells using a standard method [29], and plasmids were introduced into BY-2 protoplasts by polyethylene glycol-mediated transformation [36]. One day after transformation, GFP fluorescence images were observed using a LSM 5 EXCITER confocal fluorescence microscope with a C-Apochromat 40×/1.2W corr (water) (Carl Zeiss, Oberkochen, Germany) as an objective lens. Images were processed using the Zeiss LSM Image Browser (Carl Zeiss Inc.) and the Adobe Photoshop Element 7.0 (Adobe Systems, San Jose, CA, USA).

### Statistical analysis

Statistical significance was determined using an unpaired Student's *t* test; *P*<0.05 indicated significance.

### Accession numbers of the genes

The accession numbers for the genes described are as follows: *SLAC1* (At1g12480), *SLAH1* (At1g62280), *SLAH2* (At4g27970), *SLAH3* (At5g24030), *SLAH4* (At1g62262), *HIN1* (AB091429), *Hsr203j* (X77136), *NtRbohD* (AF506374), *OST1* (At4g33950), *CPK21* (At4g04720), *CPK23* (At4g04740), *PR1a* (At2g14610), *AtRbohD* (At5g47910).

## Results

### Involvement of anion channels on cryptogein-induced hypersensitive responses in BY-2 cells

Pharmacological evidence suggests the importance of anion channel-mediated anion efflux in cryptogein-induced initial responses in cultured tobacco cells [14]. We therefore checked the effect of an anion channel inhibitor, DIDS, on cryptogein-induced hypersensitive responses in BY-2 cells.

**Figure 1:**
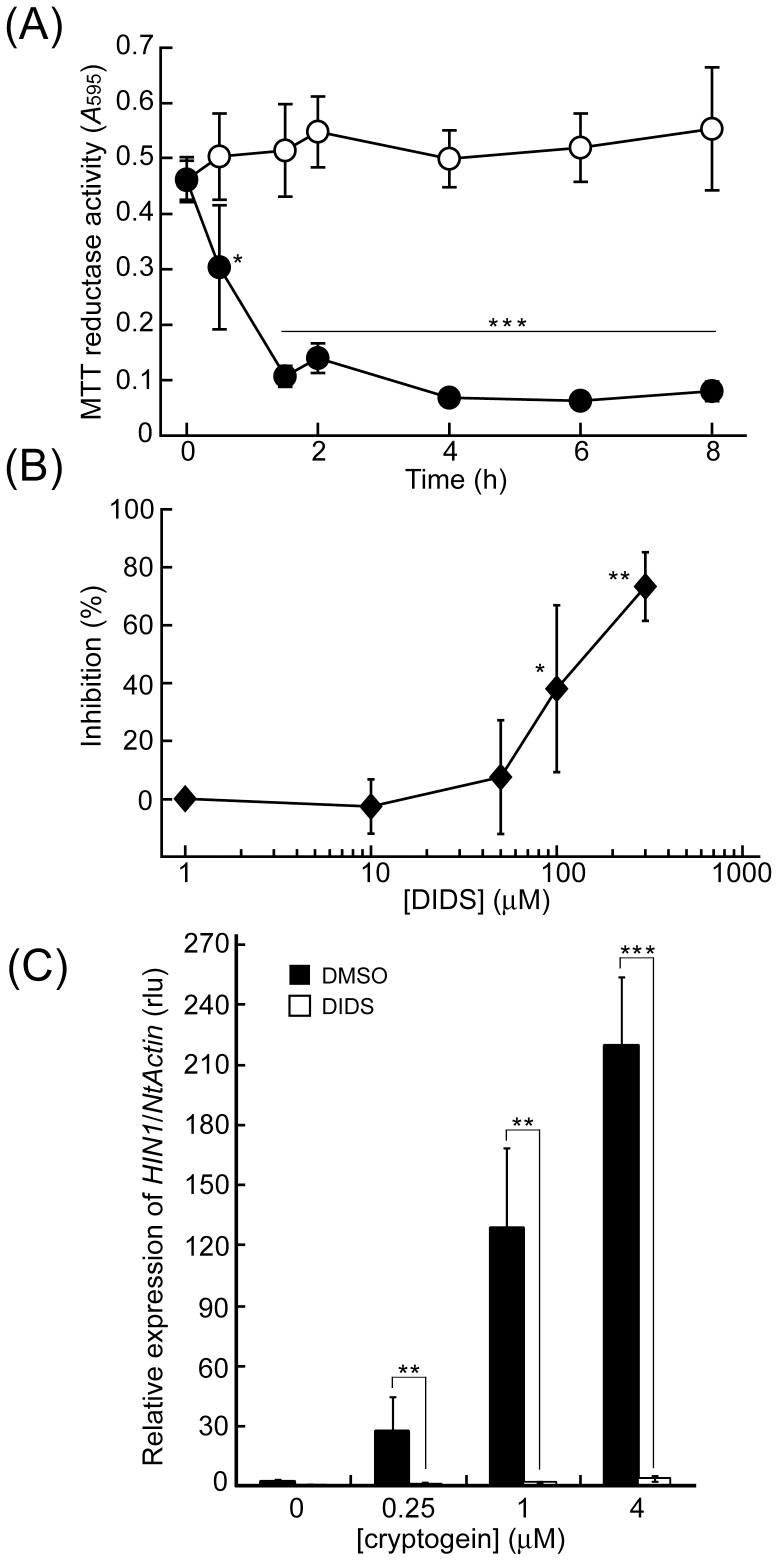
Effect of DIDS on cryptogein-induced mitochondrial dysfunction and expression of *HIN1* in BY-2 cells. (A) Cryptogein (1 µM)-induced reductions in MTT reductase activity. MTT reductase activity was used as a putative marker for mitochondrial dysfunction. Black circle: cryptogein treatment, white circle: water treatment as a control. *** *p*<0.001, significantly different from the control. (B) Inhibitory effect of DIDS on cryptogein-induced reductions in MTT reductase activity. BY-2 cells 3 h after the addition of the cryptogein elicitor (1 µM). DIDS or DMSO was added to BY-2 cells 15 min prior to the elicitor treatment. Data are the mean ± SE of three independent experiments. * *p*<0.05, ** *p*<0.005, significantly different from the control. DMSO was used as a control. (C) Effect of DIDS on cryptogein-induced *HIN1* expression. Total RNA was isolated from BY-2 cells harvested 2 h after the addition of cryptogein. DIDS (white bars) or DMSO (black bars) was added to BY-2 cells 15 min prior to the elicitor treatment. The amount of each mRNA was calculated from the threshold point located in the log-linear range of RT-PCR. The relative level of each gene in control cells at time 0 was standardized as 1. Data are the mean ± SE of three independent experiments. ** *p*<0.005, *** *p*<0.001, significantly different from the control.

Increasing evidence suggests the involvement of mitochondrial dysfunction in the induction of hypersensitive cell death in plants as well as in animals [37]. MTT reductase activity was monitored as a marker for mitochondrial function [10]. As shown in Fig. 1A, cryptogein induced a rapid reduction in MTT reductase activity in BY-2 cells in a time-dependent manner, which was significantly inhibited by DIDS (Fig. 1B). A Ser/Thr protein kinase inhibitor, K-252a also suppressed the reduction in MTT reductase activity in a dose-dependent manner (Fig. S1).

The expression of a harpin-induced gene *HIN1* has been suggested to correlate with hypersensitive responses [38]. The expression of *HIN1* was induced 5 h after the application of cryptogein (Fig. 1C), which was strongly reduced by DIDS (Fig. 1C). These results suggest that anion channels are involved in the regulation of cryptogein-induced hypersensitive responses in BY-2 cells.

### Intracellular localization of the SLAC1 protein in BY-2 cells

**Figure 2:**
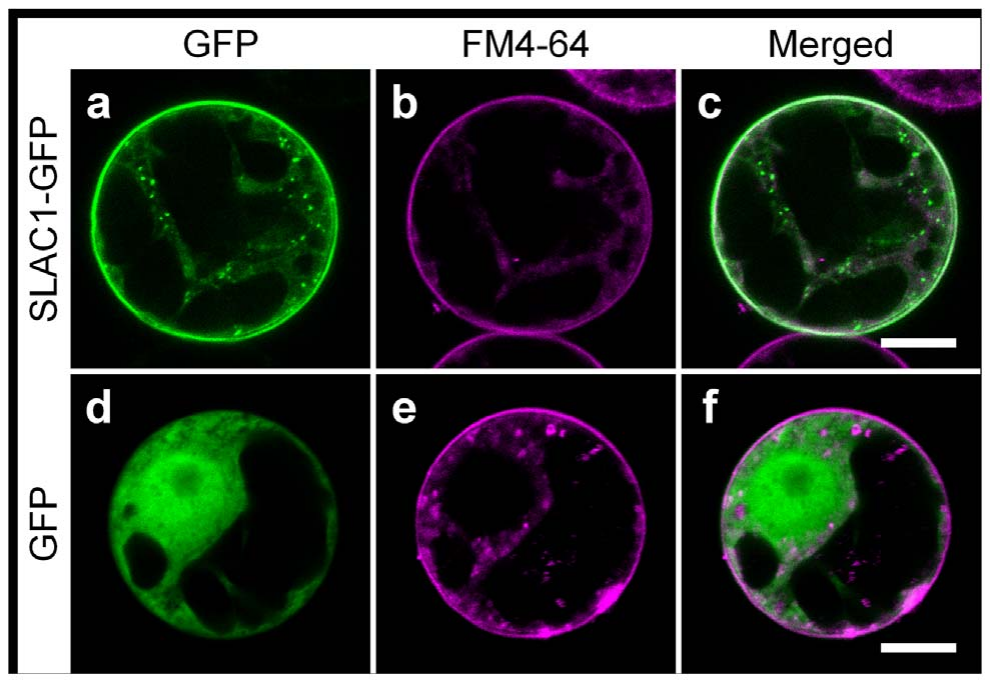
Intracellular localization of the SLAC1 protein in tobacco BY-2 cells. Confocal fluorescence images (**a**-**f**) of BY-2 protoplast expressing SLAC1-GFP (**a**–**c**) or GFP (**d–f**) stained with the fluorescent styryl membrane probe FM4-64. Fluorescence of GFP (**a** and **d**) and FM4-64 (**b** and **e**). Scale bar: 10 µm. FM4-64 was kept as a 17 mM stock solution in sterile water, and used at a final concentration of 4.25 µM. Tobacco BY-2 protoplasts were treated with FM4-64 for 10 min and washed twice with the wash buffer at room temperature to label the PM.

Arabidopsis SLAC1 is an S-type anion channel located at the plasma membrane in guard cells [20], [22]. To study intracellular localization of the SLAC1 protein expressed in BY-2 cells, we introduced the GFP construct fused to the C-terminus of SLAC1 into BY-2 protoplasts and examined its intracellular localization by confocal laser scanning microscopy. When GFP alone was expressed, it localized to the nucleus and the cytoplasm (Fig. 2; d-f). In contrast, the SLAC1-GFP fusion protein was specifically targeted to the plasma membrane; fluorescent images of SLAC1-GFP were clearly different from those of FM4-64, a marker for internal membranes (Fig. 2; a-c), indicating that SLAC1 functions at the plasma membrane in BY-2 cells as in Arabidopsis guard cells.

### Effect of SLAC1 overexpression on cryptogein-induced ion fluxes

To test possible involvement of SLAC1 in the cryptogein-induced plasma membrane anion efflux, we generated transgenic tobacco BY-2 cell lines expressing Arabidopsis *SLAC1* by means of *Agrobacterium*-mediated transformation [30]. Several transformant lines were obtained and the expression of *SLAC1* mRNA in BY-2 cells was confirmed by RT-PCR analyses (Fig. S2). Finally, four transformant lines (line No. 1–4) were selected for further investigation.

**Figure 3:**
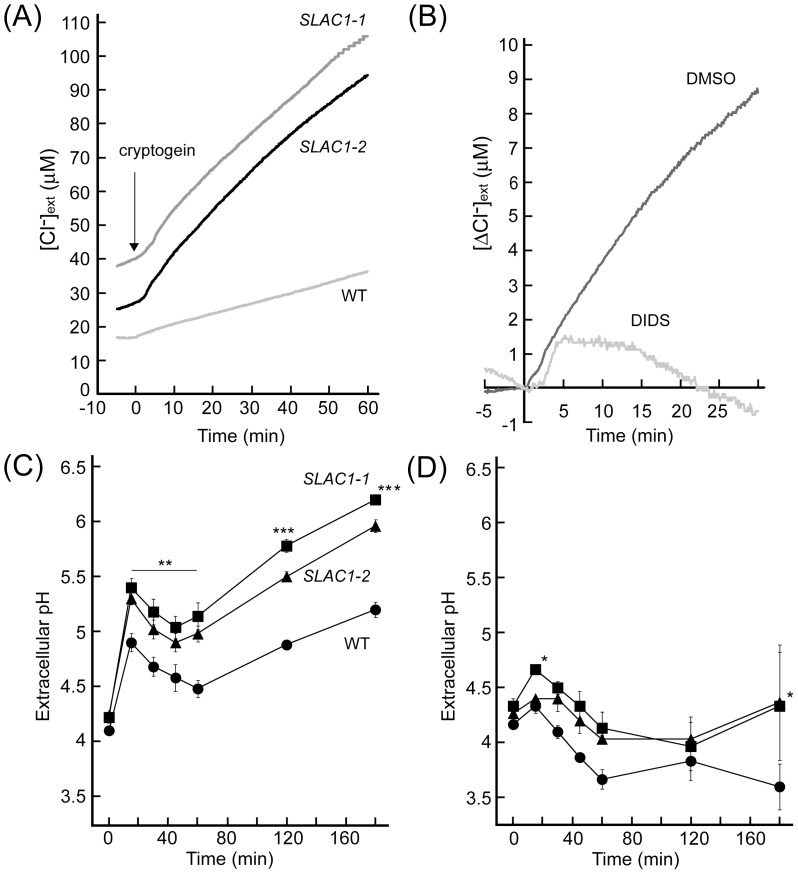
Effect of *SLAC1*-overexpression on cryptogein-induced ion fluxes. (A) Time course of cryptogein-induced [Cl^–^]_ext_ changes in BY-2 cells. BY-2 cells were treated with cryptogein (0.25 µM). (B) Effect of DIDS on cryptogein-induced [Cl^–^]_ext_ changes. DIDS (100 µM) was added to BY-2 cells 15 min prior to the elicitor (0.25 µM) treatment. DMSO was used as a control. (C) Time course of cryptogein-induced [pH]_ext_ changes. BY-2 cells were treated with cryptogein (1 µM). ** *p*<0.005, *** *p*<0.001, significantly different from the control line. (D) Effect of DIDS on cryptogein-induced [pH]_ext_ changes. DIDS (50 µM) was added to BY-2 cells 15 min prior to the elicitor treatment. DMSO was used as a control. Data are the mean ± SE of three independent experiments. * *p*<0.05, significantly different from the control line.

To confirm if SLAC1 plays a role in anion transport in BY-2 cells, we tested whether the expression levels of *SLAC1* mRNA affected anion transport activity. Extracellular Cl^−^ concentration ([Cl^−^]_ext_) was simultaneously monitored with ion-selective electrodes in the growth medium. Extracellular [Cl^−^] showed a prolonged increase after cryptogein application (Fig. 3A), which is consistent with our previous report [4]. As shown in Fig. 3A, cryptogein-induced Cl^−^ efflux was significantly higher in the *SLAC1*-overexpressing cells than in non-transgenic control cells. DIDS clearly suppressed cryptogein-induced Cl^−^ efflux (Figs. 3B), suggesting that an S-type anion channel, SLAC1, is involved in the cryptogein-induced plasma membrane Cl^−^ efflux in BY-2 cells.

Pharmacological evidence suggests the importance of Cl- efflux in cryptogein-induced initial signaling events in BY-2 cells [4], [13]. Since SLAC1 has been suggested to be involved in the regulation of Cl- efflux (Fig. 3A, B), we investigated possible involvement of SLAC1 in cryptogein-induced pH change as a marker for the initial plasma membrane signaling events [4]. Cryptogein induced biphasic slow and prolonged extracellular alkalinization (Fig. 3C), which is consistent with our previous report [4]. This elicitor–induced extracellular alkalinization was much more evident in *SLAC1* overexpressors than in non-transgenic control cells, which were severely inhibited by DIDS (Fig. 3C, D). These results suggest that SLAC1-mediated Cl- efflux modulates other ion fluxes known as initial defense signaling events in BY-2 cells.

### Involvement of SLAC1 in the regulation of cryptogein-induced biphasic ROS production

**Figure 4:**
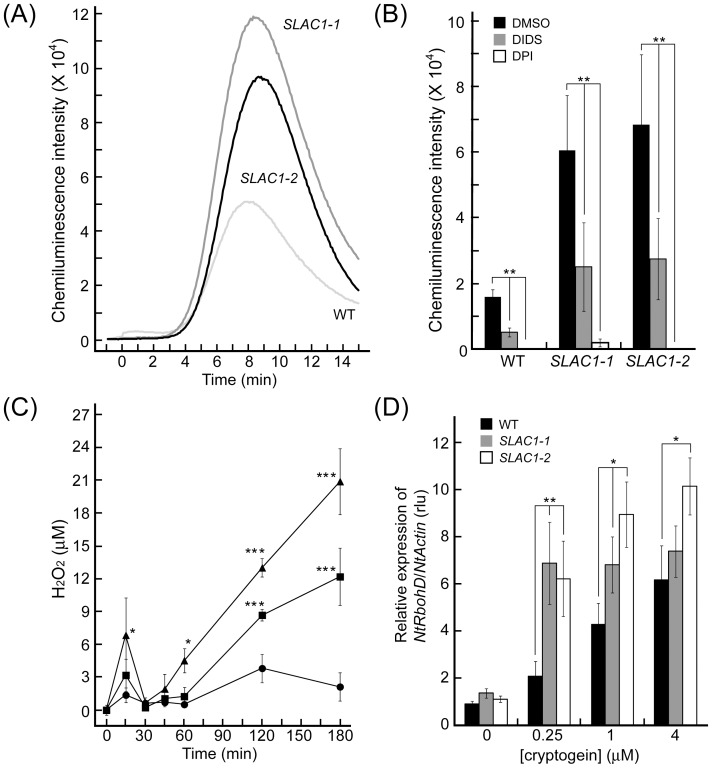
Involvement of SLAC1 in the regulation of cryptogein-induced biphasic ROS production mediated by NADPH oxidases. (A) Cryptogein (0.25 µM)-induced rapid transient ROS production within 15 min in BY-2 cells. Data is representative of three experiments (B) The effects of inhibitors on cryptogein-induced ROS production within 15 min. To quantify the effects of inhibitors on ROS production, the peak intensity of luminol chemiluminescence was compared with the control. DIDS or an NADPH oxidase inhibitor, DPI, was added to BY-2 cells 15 min prior to the elicitor treatment. DMSO was used as a control. Data are the mean ± SE of three independent experiments. ** *p*<0.005, significantly different from the control line. (C) Effect of *SLAC1*-overexpression on cryptogein-induced prolonged ROS production. BY-2 cells were treated with cryptogein (0.25 µM). Data are the mean ± SE of three independent experiments. * *p*<0.05, ** *p*<0.005, *** *p*<0.001, significantly different from the control line. (D) Quantitative expression levels of *NtRbohD* mRNAs in *SLAC1*-overexpressing cells by real-time quantitative PCR. Total RNA was isolated from BY-2 cells harvested 5 h after the addition of cryptogein at various concentrations. The amount of each mRNA was calculated from the threshold point located in the log-linear range of the RT-PCR. The relative level of each gene in the control cells at time 0. Data are the mean ± SE of four independent experiments. * *p*<0.005, ** *p*<0.005, significantly different from the control line.

Production of hydrogen peroxide (H_2_O_2_) was monitored by an assay using luminol chemiluminescence [39]. As shown in Fig. 4A, cryptogein triggered rapid ROS production within 5 min, which is consistent with our previous report [4]. The increment of ROS production was dramatically enhanced in the *SLAC1* overexpressors than the control at a low concentration of cryptogein (Fig. 4A). An NADPH oxidase inhibitor, DPI (10 µM), almost completely abolished cryptogein-induced ROS production both in *SLAC1-*overexpressors and the control (Fig. 4B), suggesting that cryptogein-triggered ROS production is predominantly mediated by NADPH oxidases [4]. DIDS clearly suppressed elicitor-induced ROS production (Fig. 4B), suggesting that overexpression of *SLAC1* leads to enhanced activation of the NADPH oxidases. Taken together, SLAC1-mediated anion efflux is suggested to affect the regulation of NADPH-oxidase activity.

Cryptogein induces biphasic (rapid/transient and slow/prolonged) ROS production in tobacco BY-2 cells [40]. Expression of a tobacco NOX homolog *NtRbohD*, encoding NADPH oxidase, is induced in response to cryptogein/elicitors or avirulent pathogens and is suggested to be essential for the slow and prolonged ROS production [40]–[43]. As shown in Fig. 4C and D, the time course of cryptogein-induced prolonged ROS production and expression of *NtRbohD* correlated well, and both of them were significantly enhanced in the *SLAC1-*overexpressors in comparison with the control at a low concentration of cryptogein. DIDS severely inhibited cryptogein-induced prolonged ROS production (data not shown) as the rapid/transient ROS production (Fig. 4B). Taken together, these results suggest that SLAC1-mediated anion flux is involved in the regulation of cryptogein-induced expression of respiratory burst oxidase homologs (Rbohs) and NADPH oxidase-mediated ROS production.

### Involvement of SLAC1 in the regulation of cryptogein-induced defense gene expression and hypersensitive cell death

Rbohs have been suggested to be involved in ROS production and the regulation of hypersensitive cell death in Arabidopsis and rice [44]–[46]. To further characterize the role of SLAC1 in the downstream signaling pathway elicited by cryptogein, we analyzed the induction of defense-related genes and hypersensitive cell death in the *SLAC1-*overexpressors.

**Figure 5:**
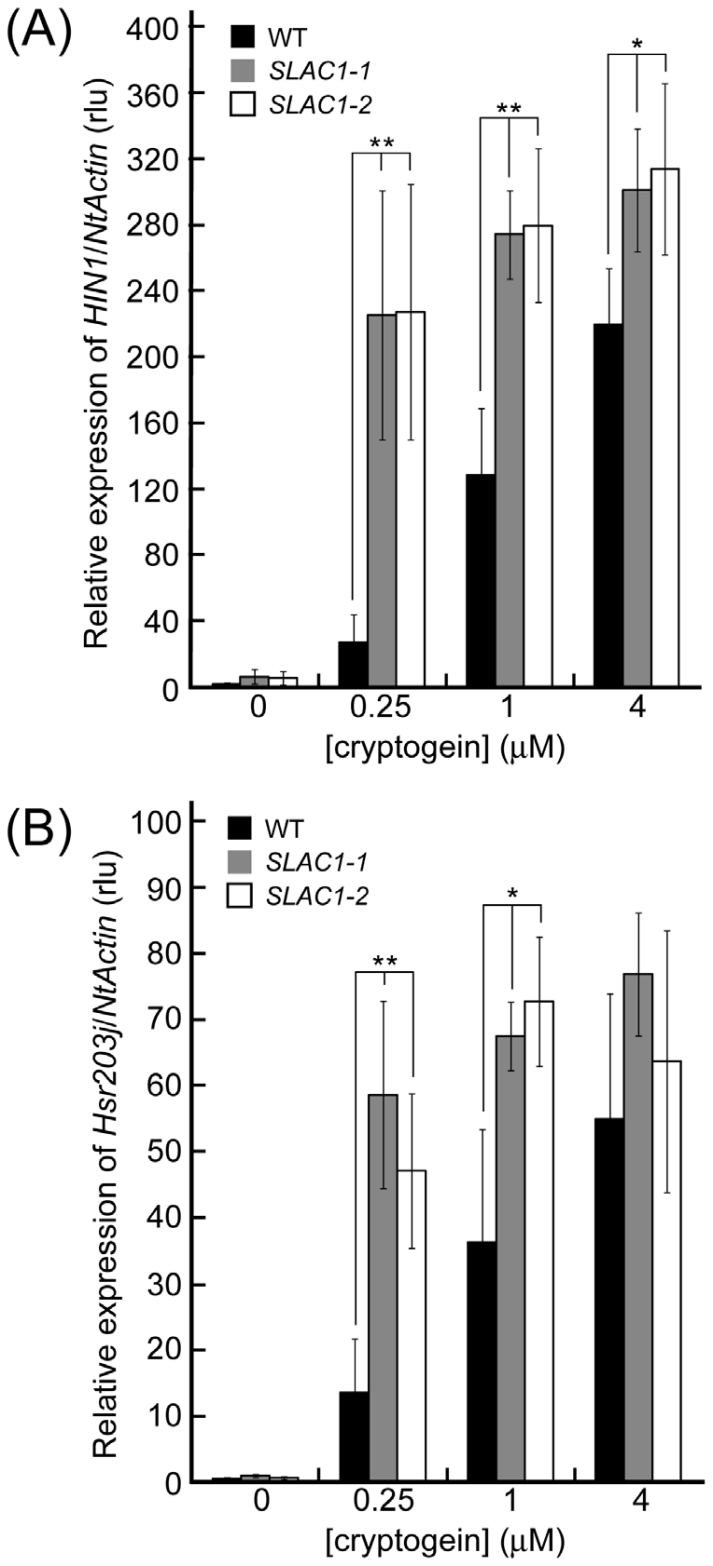
Effect of *SLAC1*-overexpression on cryptogein-induced defense gene expressions. (A and B) Cryptogein-induced the expression of *HIN1* in a dose-dependent manner (A) and *Hsr203j* (B) in *SLAC1*-overexpressing cells. The amount of each mRNA was calculated from the threshold point located in the log-linear range of the RT-PCR. The relative level of each gene in the control cells at time 0. Total RNA was isolated from BY-2 cells harvested 5 h after the addition of cryptogein at various concentrations. Data are the mean ± SE of three independent experiments. * *p*<0.05, ** *p*<0.005, significantly different from the control line.

Hypersensitive-related (*hsr*) gene, *Hsr203J*, that encodes a serine hydrolase with esterase activity, is postulated to regulate either the establishment or limitation of cell death [47]. The expression of *HIN1* is correlated with hypersensitive responses [38]. The expression of *HIN1* and *Hsr203J* were induced 5 h after the application of cryptogein in non-transgenic cells (Fig. 5A, B). At lower concentration range of the elicitor, the expression of both *HIN1* and *Hsr203J* were significantly higher in the *SLAC1*-overexpressors than in the control. At higher concentrations of cryptogein (4 µM), the expression of the genes was almost comparable between the overexpressors and the control (Fig. 5A, B).

**Figure 6:**
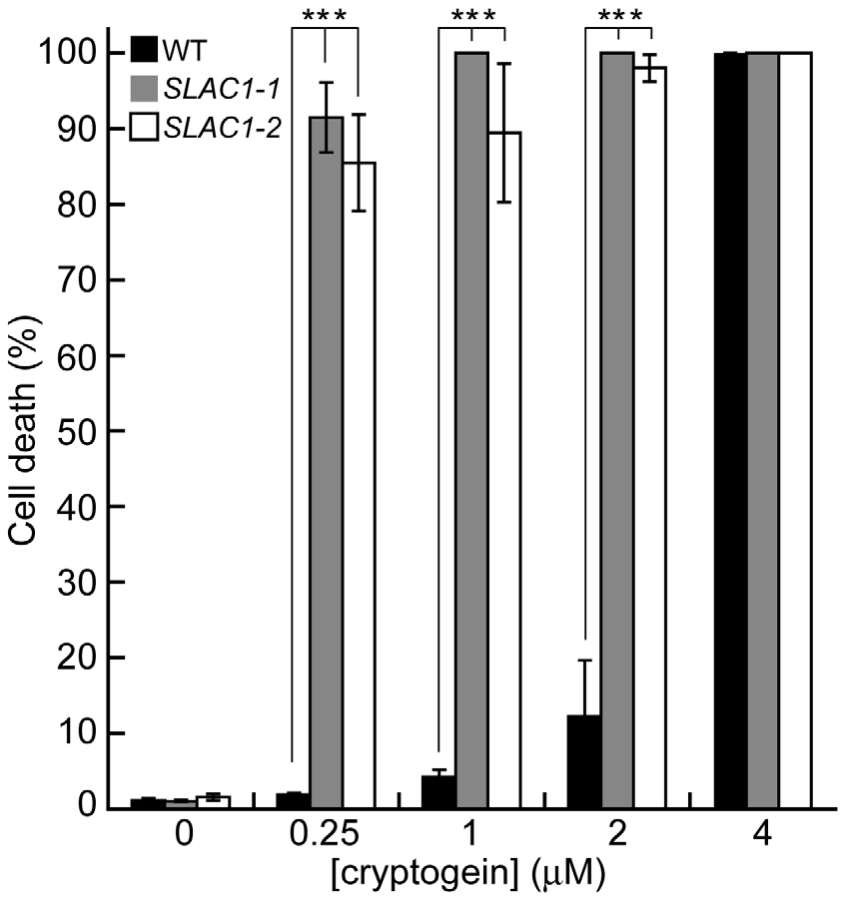
Effect of *SLAC1*-overexpression on cryptogein-induced hypersensitive cell death. Cryptogein-induced cell death in a dose-dependent manner in *SLAC1*-overexpressing cells. BY-2 cells 24 h after the addition of the cryptogein elicitor at various concentrations. Evans blue staining was applied to quantify the level of cryptogein-induced cell death. Data are the mean ± SE of five independent experiments. **** *p*<0.001, significantly different from the control line.

Hypersensitive cell death as a form of programmed cell death is a crucial event to prevent the spread of biotrophic pathogens [12]. Anion efflux has been suggested to be involved in the regulation of cryptogein-induced hypersensitive cell death in cultured tobacco cells [14]. Evans blue staining was applied to quantify the levels of cell death [40]. As shown in Fig. 6, hypersensitive cell death was much more evident in the *SLAC1-*overexpressors than that of the control cells, and the levels of cell death corresponded with the expression levels of *SLAC1*. Overall, these results indicate that the overexpression of *SLAC1* led to enhance the sensitivity to cryptogein to induce hypersensitive cell death.

## Discussion

Anion efflux has been suggested to play crucial roles in plant defense responses [4], [13], [14], [48]. However, the molecular bases for anion efflux and its regulation in immune responses remain largely unknown. The present functional characterization of the *SLAC1* overexpressors and pharmacological analyses suggest that an S-type anion channel, SLAC1, is specifically activated in response to cryptogein, and involved in the regulation of cryptogein-induced Cl^−^ efflux across the plasma membrane, defense-related gene expression, induction of hypersensitive cell death as well as NADPH oxidase-mediated biphasic ROS production in BY-2 cells.

Increasing evidence suggests the importance of mitochondria in the induction of hypersensitive cell death in defense signaling pathways [10]. We here showed that cryptogein triggered reduction in the mitochondrial reductase activity prior to the induction of hypersensitive cell death in tobacco BY-2 cells (Figs. 1A and 6), suggesting the possible involvement of mitochondrial dysfunction on the induction/regulation of the programmed cell death.

The present results indicate that cryptogein-induced mitochondrial dysfunction requires the plasma membrane anion effluxes and protein phosphorylation (Figs. 1B and S1). The anion channel activity of SLAC1 is regulated by protein phosphorylation and has been suggested to be phosphorylated by several protein kinases, such as OST1, CPK21, and CPK23, which are key regulators in the stomatal movement of Arabidopsis guard cells [49]–[52]. Recently, some Ca2+-regulated protein kinases, such as calcium-dependent protein kinases (CDPK/CPK) and CBL-interacting protein kinases (CIPK), have been shown to be involved in the regulation of a variety of immune responses including initial events in many plant species [10], [53], [54]. SLAC/SLAH family anion channels may be phosphorylated by some of these protein kinases, and the phosphorylation may be involved in the activation of elicitor-triggered anion effluxes to regulate the induction of mitochondria-mediated hypersensitive cell death.

SLAC1-mediated anion efflux plays a key role in the stomatal closure elicited by environmental stimuli, such as ABA, ozone, and CO2 in Arabidopsis guard cells [20]–[22]. However, the interrelationship among SLAC1-mediated anion efflux, transcriptional regulation of a variety of genes and ROS production as important signalling events to transmit precise initial responses to downstream pathways remain mostly unknown. We here showed that the overexpression of *SLAC1* enhanced cryptogein-induced expression of defense-related genes and *NtRbohD*, which is involved in the enzymatic ROS production (Figs. 4D and 5). Cryptogein-induced *HIN1* expression was severely inhibited by an anion channel blocker, DIDS (Fig. 1C), indicating that expression of these genes are regulated by cryptogein-induced SLAC1-mediated plasma membrane anion efflux. Anion channel-mediated anion efflux is one of the key factors in membrane depolarization, which may affect gene expression and plant immunity [55].

The present results suggest that SLAC1-mediated anion efflux is activated by cryptogein and positively modulates cryptogein-triggered NADPH oxidase/Rboh-mediated biphasic ROS production (Fig. 4D) as well as hypersensitive cell death (Fig. 6). Rboh-mediated ROS production has been suggested to be involved in the regulation of hypersensitive cell death [44]–[46]. SLAC1-mediated anion efflux activated by cryptogein may act upstream of activation of Rbohs to regulate downstream defense responses including the programmed cell death.

The cryptogein-induced rapid/transient ROS production within minutes mediated by NADPH oxidase [4] was dramatically enhanced in the *SLAC1*-overexpressors than the control (Fig. 4A, B). Interestingly, the basal expression level of *NtRbohD* and the basal ROS level before the elicitation in the *SLAC1* overexpressors were comparable to those in the control (Fig. 4A, D), suggesting that overexpression of *SLAC1* may lead to enhanced activation of NADPH oxidases by its posttranslational regulation. Rbohs are synergistically activated by direct binding of Ca2+ to their EF-hand regions and phosphorylation mediated by Ca2+−activated protein kinases [53], [56]–[59]. Ca2+ influx or activation of Ca2+−activated protein kinases may be regulated by the plasma membrane potential [60]. Interrelationship among these plasma membrane signaling events mediated by anion efflux should be an important subject for future studies.

To test whether SLAC1 has a role in other defense signaling pathways, we generated the *SLAC1*-overexpressing plants (Fig. S3) and investigated the responses triggered by flg22, a typical microbe/pathogen-associated molecular pattern (MAMP/PAMP), which does not induce hypersensitive cell death [61]. As shown in Figs. S4 and S5, flg22 induced ROS production and expression of defense marker genes *PR1a* and *AtRbohD* in Arabidopsis seedlings, which were comparable between the control and the *SLAC1*-overexpressor. These results appear to be consistent with the recent report that the membrane potential change triggered by flg22 as well as another typical MAMP, elf18 was not affected by some anion channel inhibitors including DIDS, or by a T-DNA insertional mutation in *SLAC1* or *SLAH3*, a homolog of *SLAC1* expressed in mesophyll cells [60]. The SLAC/SLAH family may not play a major role in the major MAMP signaling pathway in Arabidopsis seedlings.

In stomatal guard cells, the SLAC/SLAH anion channels play a key role as a downstream effector in the cell volume regulation [19], [20], [22], which appear to be distinct from their roles in signaling, and thus flg22-induced stomatal closure was impaired in the *slac1* mutant [62]. In Arabidopsis cultured cells, pharmacological analyses suggested that R-type anion channels have key roles in cell death and ROS production triggered by the non-specific plant pathogen *Xanthomonas campestris* [48]. Taken together, roles of various anion channels may vary among cell types and triggering signals/types of plant-pathogen interactions.

Finally, we checked the effect of cryptogein in Arabidopsis seedlings. Unlike tobacco, cryptogein scarcely induced hypersensitive responses including expression of defense marker genes in Arabidopsis (data not shown), which was consistent with a previous report [63]. Overall, the present findings suggest that cryptogein-triggered defense responses in tobacco BY-2 cells provide a unique model system for understanding the roles of S-type anion channels and SLAC/SLAH proteins in hypersensitive responses and plant immunity. The exact correlation between the levels of SLAC/SLAH proteins and the plasma membrane anion efflux remains to be evaluated. Loss-of-function experiments using *Ntslac/slah*-suppressed cell lines should be important to further unveil the roles of the SLAC/SLAH proteins in tobacco cells.

## Conclusions

**Figure 7:**
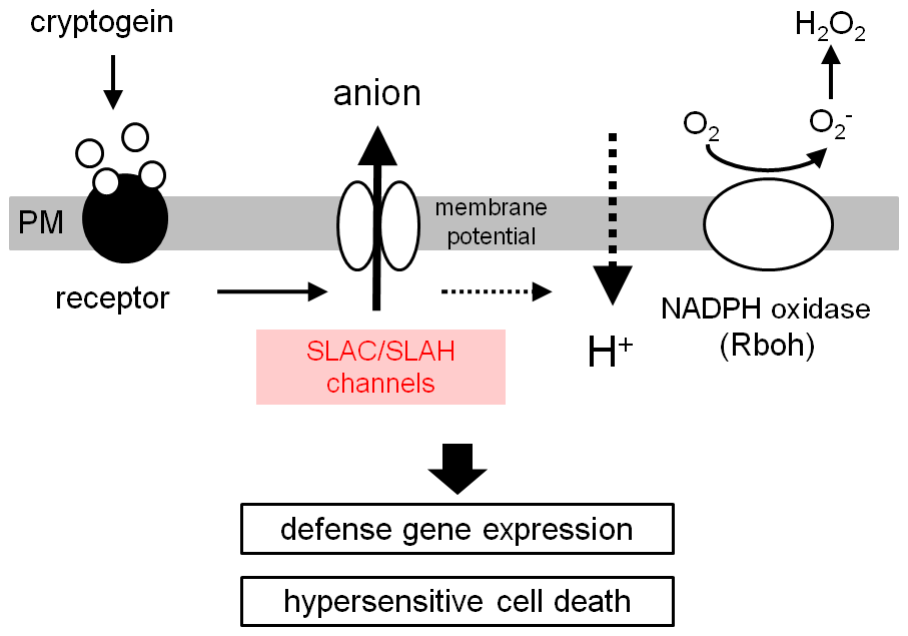
A hypothetical model for cryptogein-induced anion efflux, ROS production and hypersensitive response in tobacco BY-2 cells. Unbroken and broken arrows indicate established and hypothetical links, respectively.

The present study suggests that SLAC/SLAH anion channel(s) is involved in the regulation of the plasma membrane Cl^−^/anion efflux to modulate not only the plasma membrane signaling events such as other ion fluxes and NADPH oxidase-mediated ROS production but also the signaling pathway(s) to regulate gene expression and hypersensitive cell death triggered by cryptogein in tobacco BY-2 cells (Fig. 7). These findings shed light on the novel aspects of the roles of SLAC/SLAH anion channels and the defense/immune signaling network in plant cells.

## Supporting Information

Figure S1 **Effect of a Ser/Thr protein kinase inhibitor, K-252a, on cryptogein-induced mitochondrial dysfunction in tobacco BY-2 cells.** Cells 3 h after the addition of the cryptogein (1 µM). K-252a or DMSO was added to the cells 15 min prior to the elicitor treatment. Data are the mean ± SE of three independent experiments. ** *p*<0.005, *** *p*<0.001, significantly different from the control. DMSO was used as a control.
(TIF)

Figure S2 **Expression levels of** ***SLAC1*** **mRNA in 4 independent overexpressing lines of BY-2 cells.** First strand cDNA was synthesized from total RNA extracted from each cell lines and amplified indicated cycles by RT-PCR as described in the materials and methods. *EF1α* cDNA was used as a control DNA. PCR products were analyzed by agarose gel electrophoresis.
(TIF)

Figure S3 **Expression levels of** ***SLAC1*** **mRNA in the overexpressing lines of Arabidopsis.** First strand cDNA was synthesized from total RNA extracted from Arabidopsis seedlings and amplified indicated cycles by RT-PCR as described in the materials and methods. *EF1α* cDNA was used as a control. PCR products were analyzed by agarose gel electrophoresis. WT; wild-type (Col-0), OX; *SLAC1*-overexpressor.
(TIF)

Figure S4 **Effect of** ***SLAC1*** **-overexpression on flg22-induced ROS production in Arabidopsis.** Flg22-induced ROS production in a dose-dependent manner in *SLAC1*-overexpressing Arabidopsis seedlings. Data are the mean ± SE of three independent experiments. The Ws-0 accession of Arabidopsis does not express a functional flg22 receptor [64], and this accession was used as a negative control. Circle; *SLAC1* overexpressor, Square; wild-type (Col-0), Triangle; negative control (Ws-0).
(TIF)

Figure S5 **Effect of** ***SLAC1*** **-overexpression on flg22-induced gene expressions in Arabidopsis.** (A and B) Time course of flg22 (1 µM)-induced the expression of *PR1a* (A) and *AtRbohD* (B) in *SLAC1*-overexpressing Arabidopsis seedlings. The amount of each mRNA was calculated from the threshold point located in the log-linear range of the RT-PCR. The relative level of each gene in the control (Col-0) at time 0. Total RNA was isolated from Arabidopsis seedlings harvested at the indicated times after the addition of flg22. Data are the mean ± SE of three independent experiments.
(TIF)

File S1 **Supporting materials & methods.**
(DOC)
