# Supplementary figures and images for: An S-Type Anion Channel SLAC1 Is Involved in Cryptogein-Induced Ion Fluxes and Modulates Hypersensitive Responses in Tobacco BY-2 Cells
Source: PLoS One. 2013 Aug 12;8(8):e70623. doi: 10.1371/journal.pone.0070623 (PMC3741279; doi:10.1371/journal.pone.0070623)

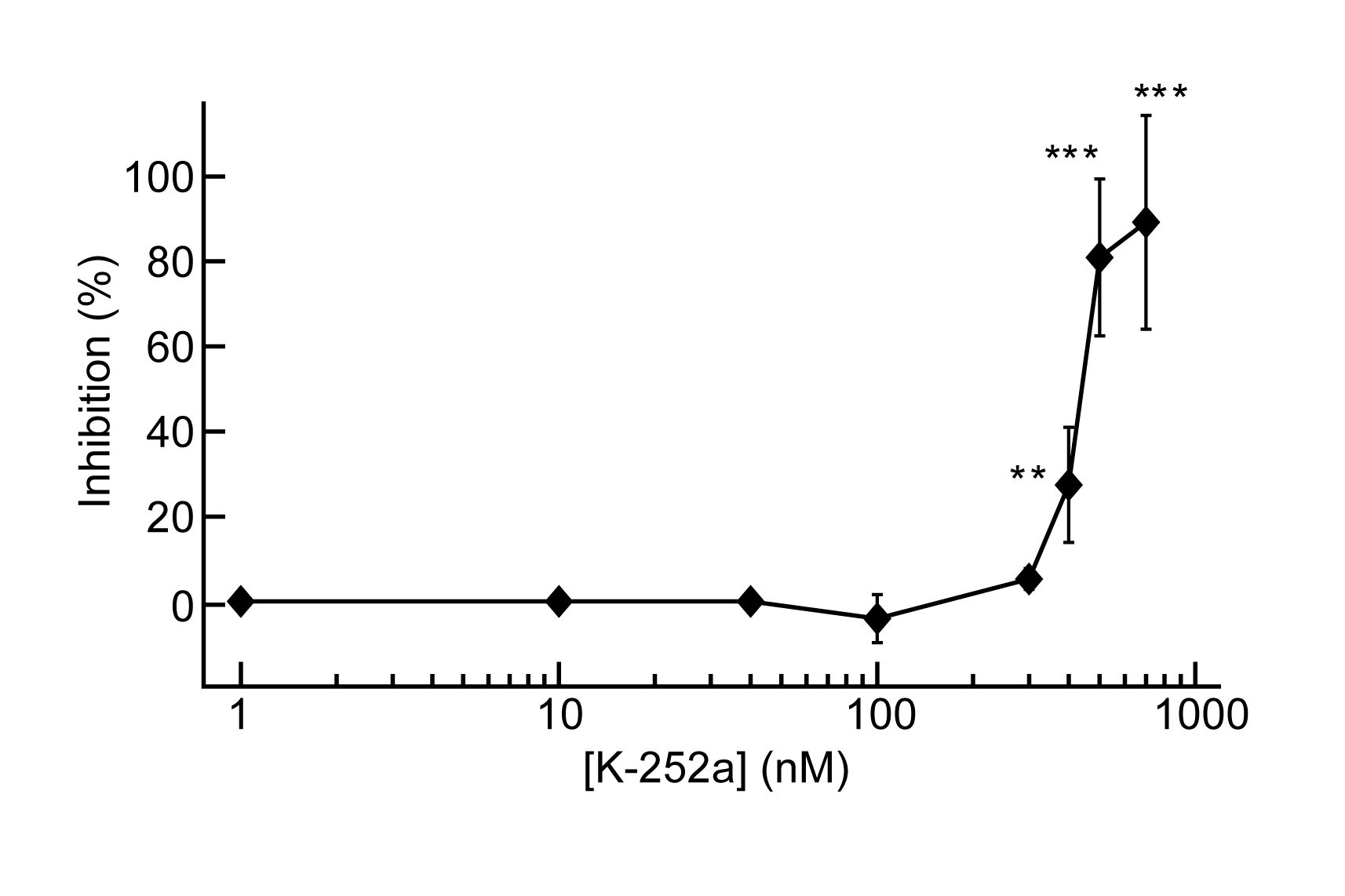

Supplement: Figure S1 — Effect of a Ser/Thr protein kinase inhibitor, K-252a, on cryptogein-induced mitochondrial dysfunction in tobacco BY-2 cells. Cells 3 h after the addition of the cryptogein (1 µM). K-252a or DMSO was added to the cells 15 min prior to the elicitor treatment. Data are the mean ± SE of three independent experiments. ** p<0.005, *** p<0.001, significantly different from the control. DMSO was used as a control. (TIF) [file pone.0070623.s001.tif]

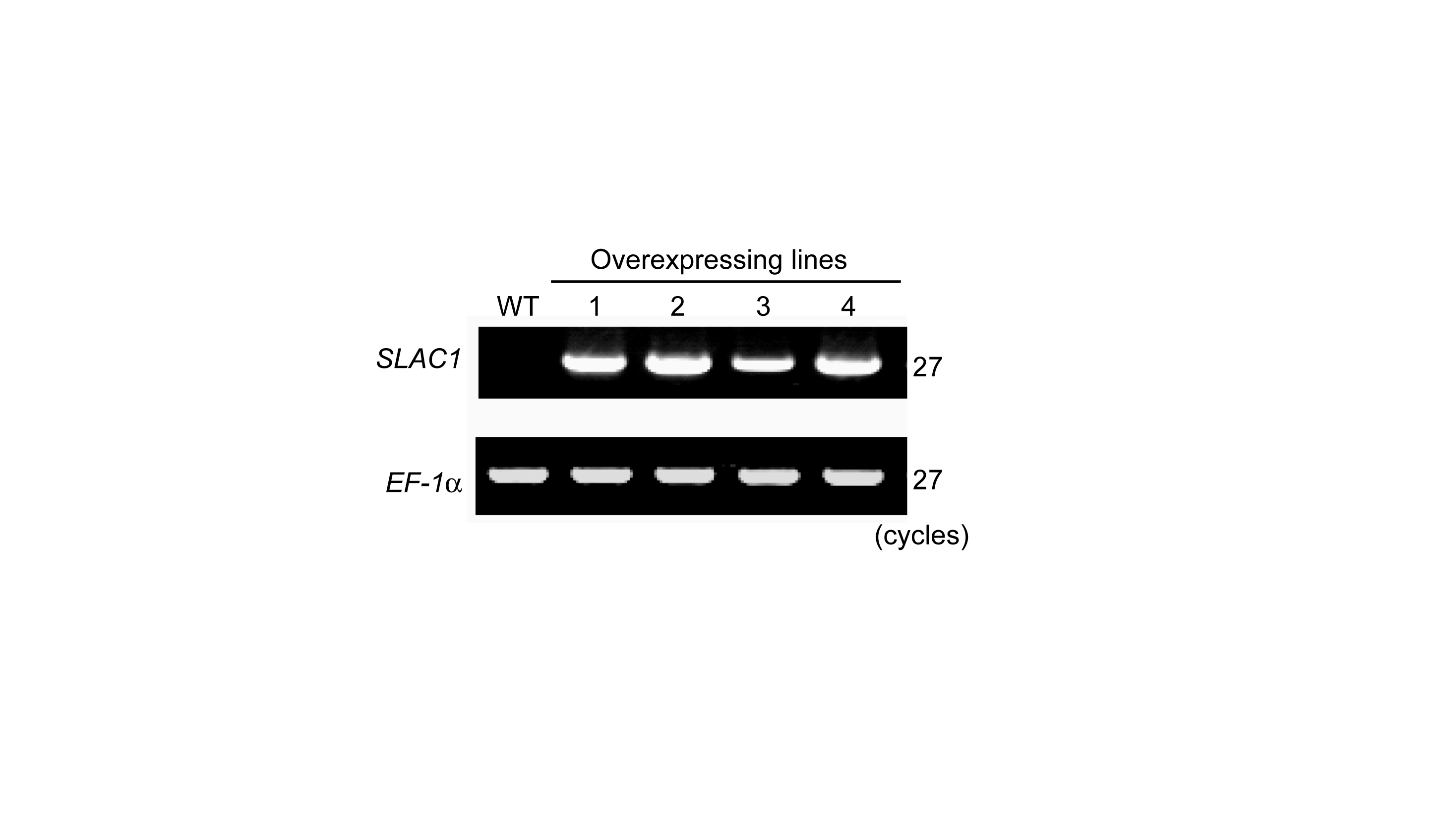

Supplement: Figure S2 — Expression levels of SLAC1 mRNA in 4 independent overexpressing lines of BY-2 cells. First strand cDNA was synthesized from total RNA extracted from each cell lines and amplified indicated cycles by RT-PCR as described in the materials and methods. EF1α cDNA was used as a control DNA. PCR products were analyzed by agarose gel electrophoresis. (TIF) [file pone.0070623.s002.tif]

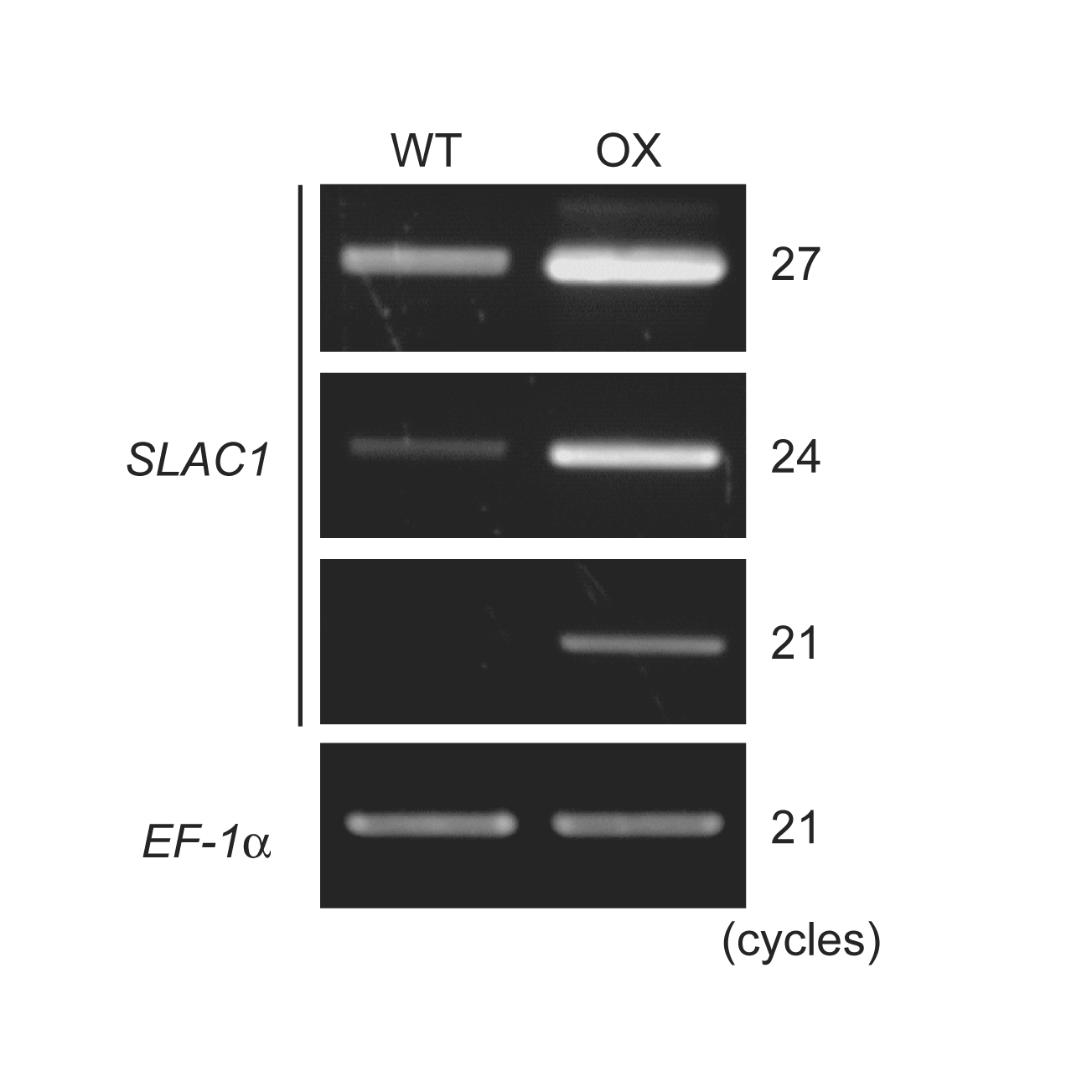

Supplement: Figure S3 — Expression levels of SLAC1 mRNA in the overexpressing lines of Arabidopsis. First strand cDNA was synthesized from total RNA extracted from Arabidopsis seedlings and amplified indicated cycles by RT-PCR as described in the materials and methods. EF1α cDNA was used as a control. PCR products were analyzed by agarose gel electrophoresis. WT; wild-type (Col-0), OX; SLAC1-overexpressor. (TIF) [file pone.0070623.s003.tif]

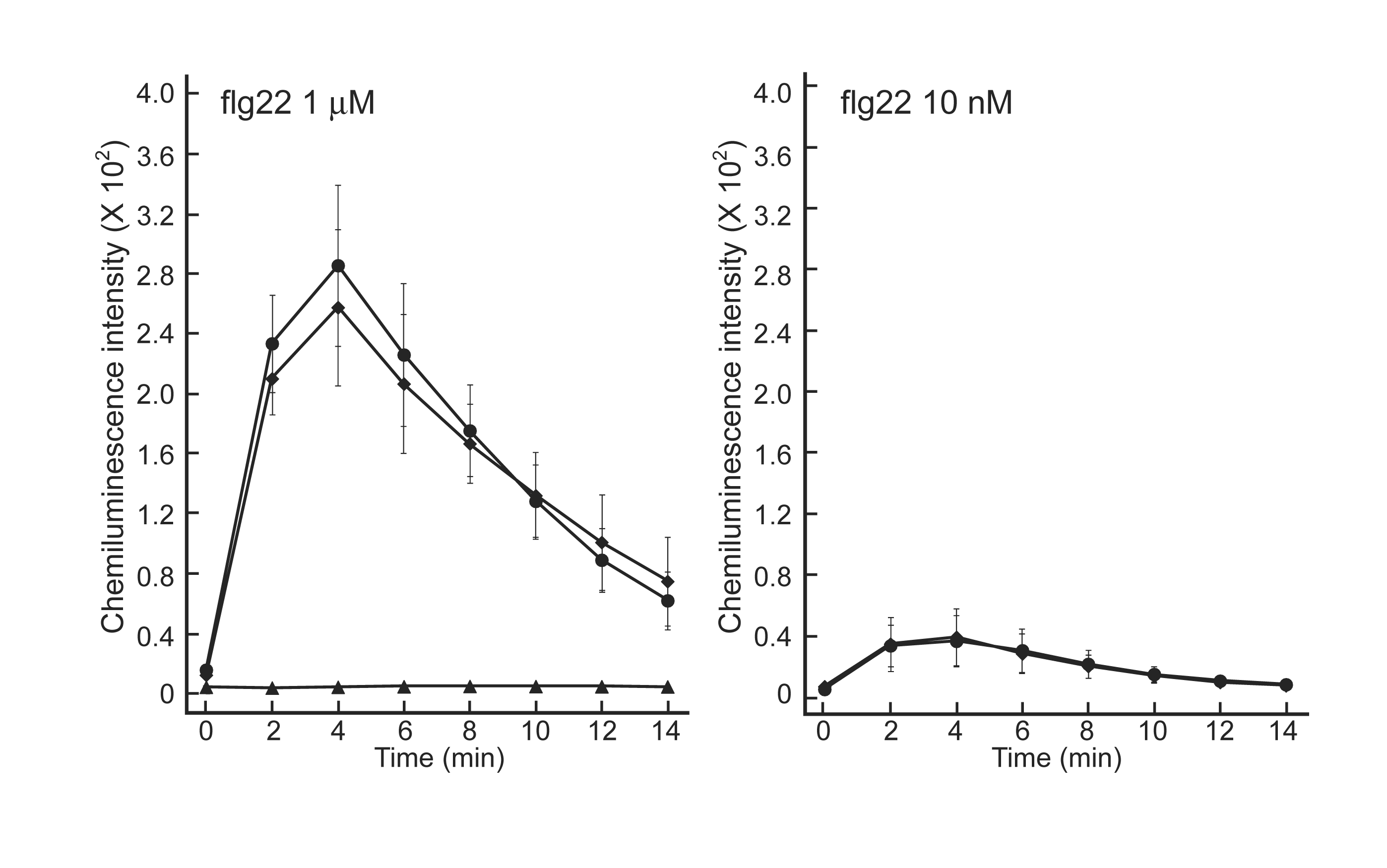

Supplement: Figure S4 — Effect of SLAC1 -overexpression on flg22-induced ROS production in Arabidopsis. Flg22-induced ROS production in a dose-dependent manner in SLAC1-overexpressing Arabidopsis seedlings. Data are the mean ± SE of three independent experiments. The Ws-0 accession of Arabidopsis does not express a functional flg22 receptor [64], and this accession was used as a negative control. Circle; SLAC1 overexpressor, Square; wild-type (Col-0), Triangle; negative control (Ws-0). (TIF) [file pone.0070623.s004.tif]

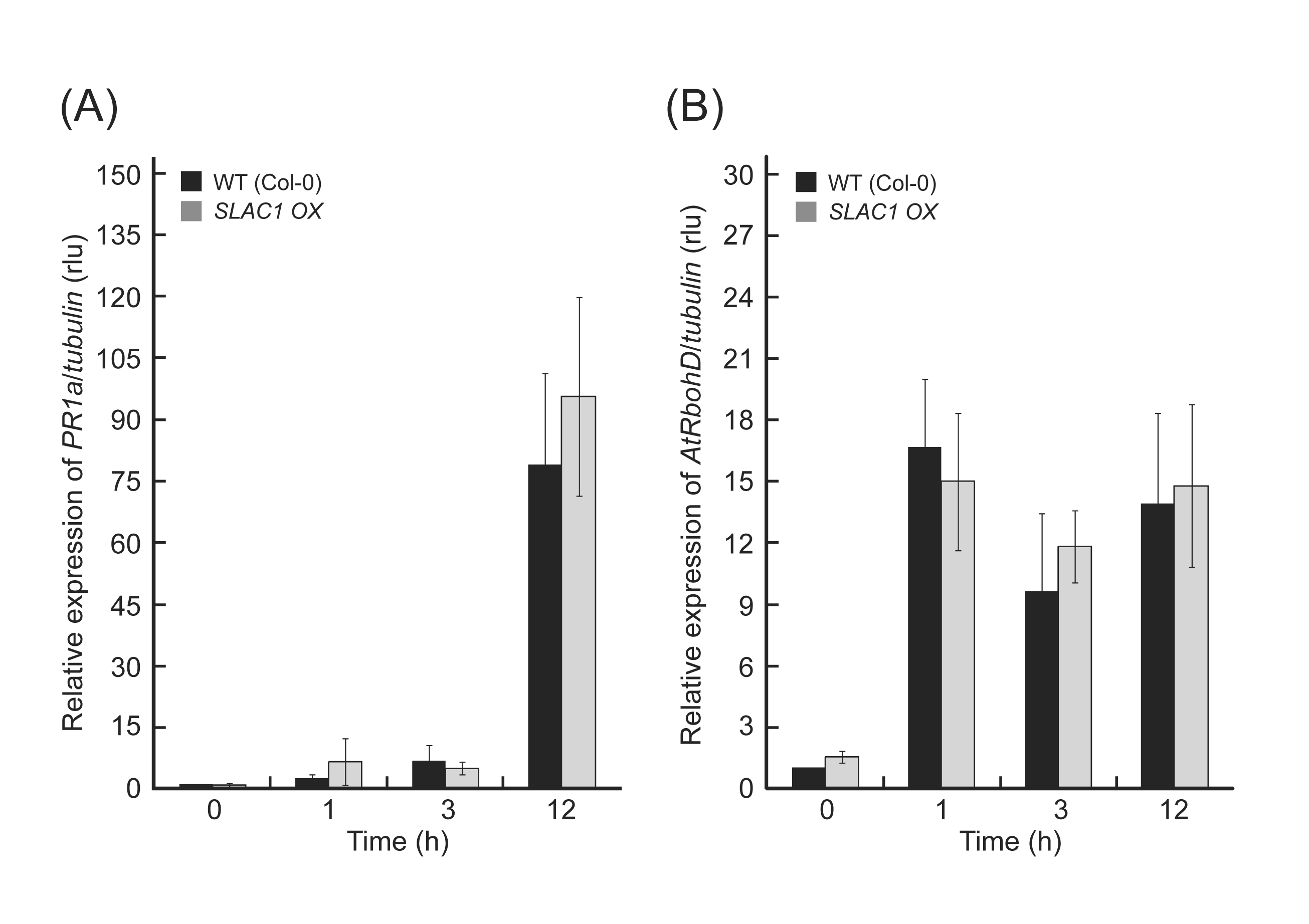

Supplement: Figure S5 — Effect of SLAC1 -overexpression on flg22-induced gene expressions in Arabidopsis. (A and B) Time course of flg22 (1 µM)-induced the expression of PR1a (A) and AtRbohD (B) in SLAC1-overexpressing Arabidopsis seedlings. The amount of each mRNA was calculated from the threshold point located in the log-linear range of the RT-PCR. The relative level of each gene in the control (Col-0) at time 0. Total RNA was isolated from Arabidopsis seedlings harvested at the indicated times after the addition of flg22. Data are the mean ± SE of three independent experiments. (TIF) [file pone.0070623.s005.tif]
