# Supplementary material for: An S-Type Anion Channel SLAC1 Is Involved in Cryptogein-Induced Ion Fluxes and Modulates Hypersensitive Responses in Tobacco BY-2 Cells
Source: PLoS One. 2013 Aug 12;8(8):e70623. doi: 10.1371/journal.pone.0070623 (PMC3741279; doi:10.1371/journal.pone.0070623)
Supplement: File S1 — Supporting materials & methods. (DOC) [file pone.0070623.s006.doc]

**Supplemental Materials and Methods**

**Plant Material**

*SLAC1* cDNA was cloned into a Ti-based vector pBI121 downstream of a CaMV *35S* promoter and transgenic Arabidopsis plants overexpessing *SLAC1* were generated using *Agrobacterium*-mediated transformation as described in [1]. Surface-sterilized seeds of *Arabidopsis thaliana* (Col-0) were germinated on MS medium containing 0.8% agar and grown in a growth chamber under long day conditions (16 h light/8 h darkness, 22°C).

**Measurement of H_2_O_2_ in Arabidopsis seedlings**

Arabidopsis seedlings grown for 11 days were used for the ROS production assay. H_2_O_2_ was monitored using a luminol-dependent assay as follows. Leaves excised from seedlings were kept on 5 mM MES buffer (pH 8.0) containing 175 mM mannitol, 0.5 mM CaCl_2_, and 0.5 mM K_2_SO_4_ for a 3 h equilibration after infiltration of the buffer by an aspirator for 5 min. Samples were then transferred to 50 mM Tris-HCl (pH 8.0, 200 μl) in a 96-well microtiter plate. The flagellin peptide flg22 (QRLSSGLRINSAKDDAAGLAIS) [2] was synthesized and used as a pathogen/microbe-associated molecular pattern (PAMP/MAMP). H_2_O_2_-dependent chemiluminescence triggered upon the addition of flg22 and luminol (25 μl, 0.462 mM) was measured every 2 min using a luminometer (MicroLumat Plus LB96V). Relative luminescence units (RLU) were presented as the average of ten samples in each measurement. The experiments were repeated at least for three times under the same condition.

**Gene expression analysis in Arabidopsis seedlings**

Total RNA was isolated from Arabidopsis seedlings and analyzed by RT-PCR as described in the text.

**References**

1. Clough SJ, Bent AF (1998) Floral dip: a simplified method for Agrobacterium-mediated transformation of Arabidopsis thaliana. Plant J 16: 735-743.

2. Felix G, Duran JD, Volko S, Boller T (1999) Plants have a sensitive perception system for the most conserved domain of bacterial flagellin. Plant J 18: 265-276.
